# Beyond clinical care: A qualitative study on the leadership role of general practitioners in dutch interprofessional care for older adults

**DOI:** 10.1080/13814788.2026.2715197

**Published:** 2026-08-26

**Authors:** Loreen M. H. Merx, Anneke van Dijk-de Vries, Henk J. Schers, Sara Chardon-Wrenger, Marloes A. van Bokhoven

**Affiliations:** ^a^ Care and Public Health Research Institute (CAPHRI), Department of Family Medicine, Maastricht University, Maastricht, The Netherlands; ^b^ Department of Primary and Community Care, Radboud University Medical Center, Nijmegen, The Netherlands

**Keywords:** General practitioners, interprofessional collaboration, leadership in primary care, informal leadership, person-centred care

## Abstract

**Background:**

Frail older adults increasingly require coordinated, interprofessional primary care, yet leadership within these loosely organised teams is often informal and poorly understood. While general practitioners (GPs) are often seen as having a central role in leadership, little is known about how they themselves perceive and enact this role.

**Objectives:**

To explore how Dutch GPs understand and operationalise informal leadership within interprofessional primary care teams caring for frail older adults.

**Methods:**

A qualitative interview study was conducted with ten Dutch GPs using semi-structured interviews. Data were analysed inductively through thematic analysis.

**Results:**

GPs expressed ambivalence about being labelled as ‘leaders’, associating the term with hierarchy. Nonetheless, they enacted leadership through responsibilities embedded in everyday practice, including ensuring continuity of care, coordinating professionals, and maintaining oversight. Leadership was largely pragmatic and reactive: GPs often initiated collaboration and provided oversight but rarely engaged in shared goal setting or facilitating team learning. Satisfaction with current collaboration and competing priorities resulted in limited motivation to optimise interprofessional practice.

**Conclusion:**

GPs enact informal leadership primarily through responsibility and accountability for continuity and oversight rather than through strategic or explicitly shared leadership practices. Because collaboration is generally perceived as adequate, motivation to further develop team processes appears limited. More strategic elements, such as shared goal setting and proactive team learning, are less developed. Strengthening awareness of leadership roles within interprofessional teams may support more balanced responsibility-sharing and enhance care for frail older adults.

## KEY MESSAGES

- GPs enact leadership implicitly through responsibilities for continuity, oversight, and coordination, without labelling themselves as leaders.
- Satisfaction with current collaboration and competing priorities may limit motivation to optimise team processes.
- Increasing awareness of leadership opportunities could support balanced responsibility-sharing and improve care for frail older adults.

## Introduction

The ageing population, often with complex chronic care needs, is placing increasing pressure on primary care systems across Europe [1]. At the same time, care is shifting from institutions to community-based settings, with general practitioners (GPs) playing a pivotal role in coordinating, integrating, and ensuring continuity of care [2,3]. These changes have led to a substantial rise in primary care workload, compounded by shortages of qualified healthcare professionals [4]. Frailty among older adults is also linked to high healthcare utilisation [5]. Together, these factors underscore the importance of interprofessional collaboration (IPC) in primary care teams to deliver coherent, person-centred care.

Recent EU reports advocate such care, emphasising interprofessional, person-centred approaches [6]. Such care relies on flexible teams whose composition adapts to individual patient needs. However, international evidence shows that collaboration in interprofessional team meetings (ITMs) often lack structure, leadership, and patient-centred focus. Meetings are frequently unstructured, and healthcare professionals tend to overestimate their teams’ effectiveness [7]. Evidence on the effectiveness of interdisciplinary team meetings for multimorbidity is limited, with a recent systematic review reporting heterogeneous outcomes across patient groups [8,9]. Ineffective teamwork wastes scarce resources, increases costs, adds to GPs’ workload, and results in fragmented care for vulnerable older adults [10,11].

To address these inefficiencies in IPC, leadership emerges as a critical factor [11–15]. Effective leadership clarifies roles and responsibilities, fosters professional development, and establishes clear communication structures [16], all of which are linked to improved team performance and patient outcomes [17]. Recognising this, the European Forum for Primary Care identifies leadership development as a crucial key enabler of sustainable, person-centred primary care [18].

**Figure 1.**
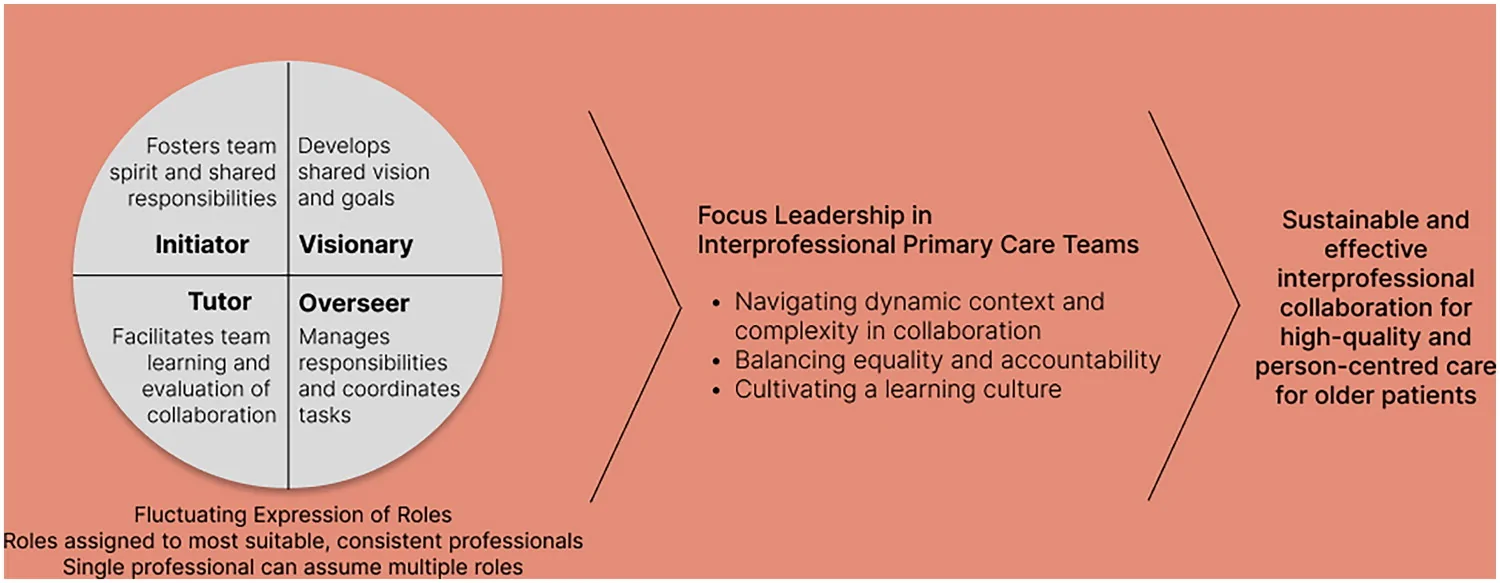
Framework for Informal Leadership in Primary Care (FIL-PC). *Source*: authors’ own work, in preprint available via DOI: 10.21203/rs.3.rs-7085121/v1.

In primary care, leadership often takes an informal form, distinct from traditional hierarchical models. Most health professionals, including GPs, do not hold formal leadership positions and may not even aspire to them [19]. Instead, leadership emerges through actions, decisions and influence. In this context, leadership can be defined as the ability of individuals to exert social influence and inspire others to achieve healthcare-related goals through personal conviction, professional standing, or shared values [19]. Building on this understanding, a Framework for Informal Leadership in Primary Care (FIL-PC, Figure 1) was developed in response to calls [20] to explore leadership beyond formal governance. The framework further conceptualises leadership as a multifaceted construct, encompassing complementary aspects such as stimulating team spirit, articulating common goals, and facilitating team learning to foster effective collaboration within loosely organised, non-managed teams.

Despite GPs often being perceived as central leaders in primary care networks, little is known about how they themselves perceive and enact this role [13,21–23]. Therefore, this study aimed to explore how GPs understand and operationalise leadership within interprofessional primary care teams.

## Methods

### Study design and context

This interview study used a qualitative descriptive design to explore practice perspectives while staying close to participants’ accounts. The study was conducted in the Netherlands, where GPs coordinate patient care within a strong primary care system and act as gatekeepers to specialist services. Care for frail older adults commonly involves interprofessional collaboration across organisations such as GPs, home care agencies, allied health professionals, and community health services. These teams typically lack a single management structure, with professionals following their own organisational rules and routines, providing an important context for examining GP leadership in primary care.

### Sampling strategy and participant recruitment

GPs were purposively sampled to obtain variation in key characteristics, including gender, years of experience, and practice characteristics (solo or group practice, and socioeconomic context of the practice area). No formal inclusion or exclusion criteria were applied beyond the requirement that participants were actively involved in interprofessional care for frail older adults. Recruitment was conducted through direct invitations, professional networks, and collaboration with a regional primary care organisation in the South of the Netherlands (ZIO). Of the GPs approached, 10 agreed to participate; none withdrew from the study.

### Data collection

Semi-structured, one-on-one interviews were conducted by a trained final-year medical student (SCW), who had received training in qualitative research and interviewing and was closely supervised throughout the study. The interviewer had no professional relationship with the participating GPs, although one participant was previously known through a clinical placement. This position encouraged open questioning and may have reduced assumptions and social desirability bias. Interviews took place at GP practices, online, or at Maastricht University, depending on participant preference. The interview guide was developed based on topics from previous studies on interprofessional leadership [7,24,25]. It included open-ended questions on GPs’ experiences, perspectives on leadership, and related challenges. The interview guide is presented in Appendix 1 (see Appendix 1).

Interviews were conducted face-to-face or online according to participant preference, lasting 27–57 min. After each interview, the verbatim transcript was discussed with experienced qualitative researchers (LM and AvD), and the interview guide and interviewing approach were refined iteratively. Data collection continued until thematic saturation was reached after eight interviews; two additional interviews confirmed saturation, yielding no new themes but providing richer insight from solo practice perspectives. All interviews were audio-recorded, transcribed verbatim, and pseudonymised.

## Data analysis

Interview transcripts were analysed using an inductive thematic analysis following Braun and Clarke’s approach [26]. Initial analysis involved iterative reading of each transcript for familiarisation with the data. SCW and LM independently open-coded all transcripts, comparing and refining codes after each interview through consensus. Analysis was iterative, with earlier transcripts revisited for consistency. SCW, LM and AvD organised codes into broader themes capturing GPs leadership patterns. The codebook is available upon request. Trustworthiness was strengthened through investigator triangulation, iterative comparative analysis, consensus discussions, and peer debriefing. Data were managed using ATLAS.ti.

### Reflexivity

During data collection, the research team was simultaneously developing the FIL-PC framework from a previous study [25]. We were aware that this could influence interpretation of the data. Regular reflexive discussions within the research team were used to critically examine interpretations and ensure transparency and analytical rigour. Participants received a findings summary for member validation, with follow-up questions where necessary. No major corrections or additions were suggested.

### Ethical considerations

The study was approved by the Maastricht University Faculty of Health, Medicine and Life Sciences Research Ethics Committee (approval number fHML-REC/2023/039). All participants provided written informed consent for participation and publication. Confidentiality was ensured through pseudonymisation of transcripts and secure data storage on a password-protected university server, accessible only to the research team.

Reporting followed the Consolidated Criteria for Reporting Qualitative Research checklist (see Appendix 2).

## Results

### Participant characteristics

The study included ten GPs, nine of whom were practice owners. They worked in solo or group practices (up to four GPs), with experience ranging from three years to over three decades. All practices were in Southern Netherlands, in neighbourhoods varying in socio-economic status and older adult population (see Table 1).

**Table 1.** Characteristics of participating general practitioners (*n* = 10).

Characteristic	*n* (%)
**Sex**	
Female	6 (60%)
Male	4 (40%)
**Age (years)**	
30–39	2 (20%)
40–49	4 (40%)
50–59	3 (30%)
60–69	1 (10%)
**Practice ownership**	
Owner	9 (90%)
Non-owner	1 (10%)
**Practice size (number of GPs)**	
Solo (1 GP)	2 (20%)
Duo (2 GPs)	2 (20%)
Group (3–4 GPs)	6 (60%)
**>10 years of experience**	8 (80%)
**Participation in interprofessional meetings**	
Active	7 (70%)
Inactive	2 (20%)
Rare	1 (10%)

Due to the 2015 Dutch Long-Term Care Act (WLZ), which restricted nursing home admission and increased primary care demand for older patients, we applied a ten-year experience cut-off. Two GPs demonstrated particular affinity with older adult care, based on interest, formal responsibilities, and experience.

All GPs had experience participating in ITMs, however, two GPs preferred ad hoc collaboration and discontinued the structured ITMs in their practice. One GP participated only occasionally in ITMs. The GPs had not received specific training in leading interprofessional teams.

### Themes emerging from interviews

The interviews revealed three interrelated themes regarding how GPs understand and enact leadership in interprofessional teams (1): ambivalence towards being seen as a leader (2), leadership embedded as a responsibility in everyday practice, and (3) limited drive to initiate change in interprofessional practice.

Notably, participants were largely aligned on these themes, with no contrasting views on the overarching patterns.

#### (1) Ambivalence towards being seen as a leader

The analysis revealed a prevalent discomfort among GPs with the label of ‘leader’ within interprofessional teams. The term ‘leader’ was associated with hierarchy, a single decision-maker, and a role where others are expected to follow. This understanding of leadership did not align with their professional identity. Instead, the data highlighted a preference for alternative terms such as ‘coordinator’ or ‘network connector’, which better reflected their emphasis on equality in IPC.

GP 06: *“I think you are certainly not alone. You are not the great I-leader who makes the rest follow. You are someone who encourages and then receives input that is valuable. So, upon which you take the next steps together.”*

GP 05: *“You need each other, especially in that frail older adult line of work. And I don’t have a monopoly on wisdom either, but I learn through the collaboration. And I hope I can help others and know that others can help me. And yes, I see myself as equal to the rest of the team.”*

Equality was widely valued. However, the analysis identified a subtle, informal power dynamic among GPs, which influenced team dynamics. Their professional authority appeared to lend additional weight to their opinions, even within teams that prioritised equality, thereby potentially shaping decision-making processes.

GP04: *“But there has to be someone… who can address people. As GPs, we can say anything. (…) If the assistant says something about it, everyone* [other team professionals] *thinks, ‘Yeah, sure… it’s fine with you, you should just be glad I even show up.’ They would of course never say that to a GP.”*

GPs acknowledged that they were implicitly expected to take the lead—particularly when no formal leader had been designated.

GP 03: [Regarding who is in the lead] *“If it has not been stated who it should be, the other organisations will think that the GP has it.”*

However, this externally attributed assumption of the GP’s inherent leadership role within interprofessional teamwork was also questioned.

GP 04: *“I would like the general practitioner to be less at the top. Because being at the top cannot be explained on the basis of knowledge. (…) It is not your merit that makes it so, but it has something to do with regulations and history.”*

#### (2) Leadership embedded as a responsibility in everyday practice

While none of the GPs explicitly identified as leaders, leadership was constantly associated with the responsibilities they carry—towards both patients and other professionals within the care network. A central aspect of this responsibility was the continuity of care. Longstanding relationships enabled GPs to contribute contextual knowledge and person-centred insights into interprofessional decision-making.

*GP 01: “The longer you are in a practice, the better your historic context of people becomes. (…) While other professionals of an interprofessional meeting fly in at the moment that problems occur… But as a GP, you have known that person for ten years already, perhaps longer.”*

A strong personal obligation to ensure tasks were completed properly emerged, stemming from a sense of being the final advocate for patients. This responsibility was deeply embedded in medical training, where GPs learned to make decisions and take accountability for their outcomes. Accountability was characterised as both a moral duty and a legal obligation, as regulations often designate GPs as the final decision-makers.

*GP 04: “It is just the way it is that many things are placed with the GP, right. And in terms of regulations, it is often the case that the GP has the last word. So, if the GP removes that hand, then a lot of things just aren’t going to happen.”*

*GP 07: “Because you have that final responsibility, it may also mean that you are more active in your role. (…) Then it is just very important that it goes well.”*

GPs’ responsibility was marked by greater accountability and more immediate consequences for patients compared to that of other care professionals.

*GP 03: “The home care workers are very good at gathering information, but their job description also includes less responsibility… So, I do understand that they think something like: ‘Well, I’ll just call the GP, because then at least, I’ve reported it, and whatever the GP does with it, that’s up to them.”*

In practice, GPs frequently are the first point of contact in complex cases, identifying needs and involving relevant professionals. In this way, GPs perceived themselves as connectors in the professional network, responsible for linking, contacting, and coordinating different actors. Beyond clinical insight, system-level knowledge—such as regulations, referral pathways, and funding structures—emerged as part of their leadership expertise.

*GP 03: “The GP is called in and then I mainly have a kind of overview: What is going on here? Is it medical, psychological, psychiatric? A healthcare problem? A logistical problem? I then try to map that out and call on all those auxiliary troops.”*

*GP 05: “We also often coordinate the collaboration. Sometimes the physiotherapist finds the occupational therapist, but usually I find the physiotherapist, and I find the occupational therapist, and then I connect them to each other from my role.”*

An oversight role was identified, ensuring the care process progressed smoothly, even without direct involvement in every task. Gaps in collaboration or missing roles frequently required intervention, reinforcing the position as the ultimate safety net for patients.

*GP 05: “I think we are the initiator when there is a problem, and the one with final responsibility for a problem. And in between the one who should have the overview, like: ‘is it going as it should?’ and then be less the executing party, which others are more.”*

*GP 10: “For a while we did not have a case manager. (…) So, then there were patients who were actually a bit left to their fate, or we had to take over again. (…) Because in the end, we are often the last, well, safety net for those patients.”*

In sum, participants’ accounts highlight that GPs experience their leadership primarily as a set of responsibilities rather than a formal role. Their leadership is expressed through bearing ultimate accountability and advocacy for patient outcomes and maintaining an overview of the interprofessional care process—balancing coordination, oversight, and direct involvement as required.

#### (3) Limited drive to initiate change in interprofessional practice

GPs’ understanding of IPC was largely shaped by their experiences with ITMs, which were the first and most frequently mentioned form of collaboration. These meetings differed in structure and frequency, with some being regular, well-organised sessions, while others adopted a more ad hoc approach to communication and collaboration. ITMs were almost exclusively case-based, with little attention given to broader team processes such as setting shared goals, clarifying responsibilities, or evaluating collaboration. Although ITMs had traditionally been organised in larger interprofessional teams, a shift towards smaller meetings was observed over time. Smaller ITMs proved more manageable and conducive to meaningful discussion (e.g. including the GP, practice nurse and community nurse or dementia case manager), whereas larger meetings, which often included additional professionals such as physiotherapists, social workers of pharmacists, were frequently perceived as repetitive or superficial.

*GP 06: “You are often discussing things that have already been discussed, only now with the large team so everyone is up to date. But in the end, everyone often already knows everything.”*

Several benefits of ITMs were identified, including relationship-building with colleagues, short communication lines, and access to different perspectives and informal consultations. ITMs also served as a forum for aligning care plans and making joint decisions. Drawbacks included time-consuming meetings, substantial organisational effort, suboptimal attendance, and infrequent opportunities for urgent cases. In cases where ITMs had been discontinued, relationships and short communication lines were maintained through intensive informal contact, both by phone and by being co-located with other professionals.

Despite these benefits, ITMs were not found to foster a strong sense of collective ownership or shared group identity. Collaboration occurred, but responsibilities often remained largely individual. Processes, such as optimising structures and routines for ongoing IPC, often failed to initiate without GP leadership. Frustration arose when these responsibilities were rarely taken up by other team members.

GP 04: (Regarding the role of the GP in maintaining structured interprofessional meetings) *“If the doctors aren’t available, then it will be called off. So, you see that if you remove that structure and do not make the effort to maintain it, (…) within a year it’s completely gone. Then nobody will remember that it ever existed.”*

There was general satisfaction with current collaboration in older adult care. GPs recognised the growing complexity of care and occasional concerns about sustainability, but improving ITMs or IPC was not seen as a priority.

*GP 05: “I actually think (…) that we are satisfied with how we are doing now. (…) Could you optimise it? Yes, undoubtedly. But in everyday pragmatics, (…) it would be 31st on my list to refine.”*

This pragmatic approach was reflected in a focus on day-to-day problem-solving, based on the shared assumption that all professionals aimed to provide good patient care.

GP02: [Regarding shared goals among professionals] “*And everyone will have the patient’s best interests at heart. In that, you do find common ground.’’*

Evaluation of teamwork occurred reactively, after issues had arisen, rather than as part of a deliberate leadership practice. Although the idea of mutual feedback was appreciated, it was seldom systematically embedded, relying instead on the expectation that colleagues would speak up when needed.

GP 03: *“I think there is an atmosphere that if something is not going well, you can address it with each other. (…) I hope that people would hold me accountable for that, at least, that is how I would do it with someone else.”*

## Discussion

This study examined how GPs understand and enact informal leadership within interprofessional primary care teams caring for frail older adults. Although GPs do not identify with the label ‘leader’, their accounts reflect a mix of responsibilities and leadership behaviours. Our results reflect the inherently hybrid nature of modern medical careers, where clinical practice coexists with informal organisational responsibilities. Discussing this through the lens of identity frameworks reveals that while GPs enact leadership behaviour, they resist the formal label to protect their primary identity as clinicians [27]. These responsibilities were primarily framed as a professional duty to ensure continuity of care and to act as a final safety net for older adults. Maintaining oversight, coordinating with other professionals, and carrying accountability for clinical decisions emerged as part of their professional role rather than formal leadership. Despite increasing demands, most GPs expressed satisfaction with current collaboration and showed little motivation for structural improvement.

### Exploring leadership roles through the FIL-PC framework

It is insightful to compare GPs’ everyday experiences of leadership with the expert-informed perspectives captured in the FIL-PC framework [25], as this helps to clarify how informal leadership is understood and enacted in practice. This comparison highlights that GPs enact predominantly pragmatic, context-driven approaches rather than strategic or formalised leadership. GPs most often aligned with the Initiator role, particularly in initiating and maintaining collaboration. Differences in how they fostered shared ownership and motivation appeared linked to individual style, working arrangements, and opportunities for informal contact. However, their tendency to avoid addressing perceived passivity in colleagues may undermine long-term team cohesion.

The Visionary and Tutor roles were rarely enacted explicitly. Shared goals were usually assumed rather than defined, and team reflection occurred mainly in response to problems rather than proactively. Descriptions of ITMs reflected this pattern: they focused on individual cases with little time for shared goal setting, collaborative agreements, or team evaluation. Small ITMs were perceived as more effective for in-depth case discussions, whereas large meetings often led to superficial updates. These practices suggest that while some aspects of the Visionary and Tutor roles –such as the preference for smaller ITMs– are present, more intentional shared goal setting and proactive team learning are largely absent. As a result, teams may overlook the longer-term leadership and development needed for sustainable IPC, becoming vulnerable when members leave or are unavailable.

In the Overseer role, GPs monitored follow-up on agreed actions and safeguarded continuity of care, though this oversight was mostly informal and reactive. Their position as the “final safety net”, grounded in legal and professional accountability, can unintentionally centralise responsibility and workload. Rather than reducing GPs’ accountability, the findings suggest a need for teams to explicitly discuss and operationalise shared responsibility. Evidence on shared care and distributed leadership shows that such practices can strengthen task allocation [28]. For instance, research demonstrates that primary care and community nurses can effectively enact leadership roles within primary care networks, offering a viable pathway towards such distributed leadership [29].

Overall, GPs primarily enact the Initiator and Overseer roles, while shared goal setting and proactive team learning remain limited. This suggests that leadership should be viewed not only as an individual task but as a distributed function that teams must consciously develop for balanced, sustainable collaboration.

### Barriers to optimising interprofessional collaboration in everyday practice

Despite evidence that integrated teams with well-distributed leadership can improve care quality and reduce crisis admissions in dementia care [30,31], GPs place less emphasis on optimising IPC. This may reflect comfort with the status quo, driven by time pressures, competing priorities, and satisfaction with current collaboration. Previous research shows that professionals often overestimate the quality of ITMs, further reducing motivation for change [7]. Although IPC is promoted in policy as a systemic solution to healthcare pressures [17,32], our findings suggest that GPs do not automatically perceive it as a meaningful solution in practice. Teams may therefore be insufficiently aware of how leadership could enhance collaboration and care delivery. To bridge current gaps in proactive care, interprofessional clinical leadership training is essential to raise awareness of the four FIL-PC roles. These competencies could support more sustainable collaboration, reduce fragmentation of care, and improve continuity and person-centred care for frail older adults [33].

### Strengths and limitations

A key strength of this study is its iterative and reflexive approach to data collection and analysis. Although the primary interviewer was a sixth-year medical student with limited qualitative and general practice experience, this novice position encouraged open questioning, reduced assumptions, and may have made participants less inclined to give socially desirable answers.

Another strength is that all participants were from South Limburg, a region with an already advanced ageing population. This context enabled the study to capture pressures and challenges of IPC and informal leadership early on, offering rich insights into organising care for frail older adults. Although this may limit direct transferability to regions with younger populations, the core challenges identified are likely relevant across many European settings where community-based older adult care is becoming a growing priority.

Although the number of interviews was relatively small, data adequacy is supported by the fact that thematic saturation was reached. Our sample included GPs with diverse working arrangements and experiences, yet remained relatively homogeneous in terms of professional background and involvement in interprofessional care for older adults. Prior evidence shows that saturation in such homogeneous samples is often achieved with a limited number of interviews [34]. This aligns with our study’s clearly defined focus on how GPs understand and operationalise leadership within interprofessional primary care teams. Given this narrow scope and the consistent patterns across interviews, saturation was likely reached efficiently.

We interviewed nine practice owners and one locum GP. Our focus on practice owners was deliberate, as their long-term presence within the community supports sustained relationships with interprofessional colleagues and enables consistent leadership within teams. The locum GP offered complementary perspectives; however, the leadership challenges specific to locum GPs are likely different and cannot be fully captured through a single interview.

Finally, this study examined the GP perspective as a necessary first step in understanding leadership dynamics within interprofessional primary care teams. Because such leadership is relational and often informal, perspectives of other team members will be essential in future research. Although GPs emphasised equality in collaboration, their structural position—through referral authority, influence on team processes and, at times, employment relationships—may complicate this perceived equality. They also noted that others often assigned leadership roles to them and assumed that stakeholders communicate openly when issues arise. It remains unclear whether stakeholders indeed ascribe leadership to GPs, feel equally able to raise concerns or experience the same sense of equality. Including stakeholder perspectives will therefore offer a more complete understanding of how leadership is enacted and how influence and responsibility are distributed across teams.

### Future research

To build upon these insights, future research should first incorporate the perspectives of other interprofessional team members (e.g. nurse practitioners). This is essential to explore whether they experience the same team equality and ascribe leadership to GPs. Second, future studies should investigate locum GPs, who likely face distinct challenges in establishing informal authority due to their transient positions. Finally, intervention studies are warranted to evaluate whether FIL-PC–based team training or reflection tools can raise awareness of underutilised leadership roles and foster proactive team learning in daily practice.

## Conclusion

This study shows that although GPs do not see themselves as ‘leaders’, they take on leadership responsibilities in interprofessional care for frail older adults. Their leadership is rooted in accountability for patient outcomes and continuity of care, leading them to initiate collaboration, coordinate professionals, and provide oversight. More strategic elements, such as defining shared goals or fostering proactive team learning, appeared to be less developed. While GPs value collaboration, most perceive limited need or capacity to structurally improve it. Raising awareness among GPs and their interprofessional partners of leadership may help foster more balanced teamwork. Emphasising shared leadership across all team members is needed to reduce over-reliance on the GP and ensure sustainable, person-centred care across primary care systems.

## Supplementary Material

Supplemental Material

Supplemental Material

## References

[1] OECD. Integrating care to prevent and manage chronic diseases: best practices in public health. Paris; 2023.

[2] Kaljouw X, Van Vliet K. Naar nieuwe zorg en zorgberoepen: de contouren. [Towards new healthcare and health professions: the contours.]. Zorginstituut Nederland; 2015.

[3] Schmalstieg-Bahr K, Popert UW, Scherer M. The role of general practice in complex health care systems. Front Med (Lausanne). 2021;8:680695. doi: 10.3389/fmed.2021.680695. 34901044 PMC8655229

[4] European Commission. Joint research centre. Healthcare workforce: new model projects EU needs up to 2071. 2024. Available from: https://joint-research-centre.ec.europa.eu/jrc-news-and-updates/healthcare-workforce-new-model-projects-eu-needs-2071-2024-12-06_en.

[5] Ilinca S, Calciolari S. The patterns of health care utilization by elderly europeans: frailty and its implications for health systems. Health Serv Res. 2015;50(1):305–320. doi: 10.1111/1475-6773.12211. 25139146 PMC4319884

[6] European Commission. The EU health workforce: trends and challenges. Brussels; 2012.

[7] Van Dongen JJJ, Van Bokhoven MA, Daniëls R, et al. Interprofessional primary care team meetings: a qualitative approach comparing observations with personal opinions. Fam Pract. 2017;34(1):98–106. doi: 10.1093/fampra/cmw106. 28122925 PMC5266085

[8] Bouton C, Journeaux M, Jourdain M, et al. Interprofessional collaboration in primary care: what effect on patient health? A systematic literature review. BMC Prim Care. 2023;24(1):253. doi: 10.1186/s12875-023-02189-0. 38031014 PMC10685527

[9] Lammila-Escalera E, Greenfield G, Barber S, et al. A systematic review of interventions that use multidisciplinary team meetings to manage multimorbidity in primary care. Int J Integr Care. 2022;22(4):6. doi: 10.5334/ijic.6473. PMC9585979 36348941

[10] Khazen M, Shalev L, Golan-Cohen A, et al. Teamwork among primary care staff to achieve regular follow-up of chronic patients. Ann Fam Med. 2025;23(2):100–107. doi: 10.1370/afm.240176. 40127969 PMC11936351

[11] Zijl ALV, Vermeeren B, Koster F, et al. Interprofessional teamwork in primary care: the effect of functional heterogeneity on performance and the role of leadership. J Interprof Care. 2021;35(1):10–20. doi: 10.1080/13561820.2020.1715357. 32053403

[12] Van Drielen E, De Vries AW, Ottevanger PBN, et al. Beter multidisciplinair overleg past bij betere zorg. [Better multidisciplinary meetings align with better care.] Ned Tijdschr Geneeskd. 2012;156: a 4856. 23134746

[13] Grol SM, Molleman GRM, Kuijpers A, et al. The role of the general practitioner in multidisciplinary teams: a qualitative study in elderly care. BMC Fam Pract. 2018;19(1):40. doi: 10.1186/s12875-018-0726-5. 29523092 PMC5845178

[14] Borrill C, West M, Shapiro D, et al. Team working and effectiveness in health care. Br J Healthcare Manag. 2000;6(8):364–371. doi: 10.12968/bjhc.2000.6.8.19300.

[15] Halas G, Singh J, Cardenas E, et al. Team leadership for interprofessional collaborative practice in primary care: a scoping review. Ann Fam Med. 2023;21(Supplement 3):5367 doi: 10.1370/afm.22.s1.5367.

[16] Grol R, Ouwens M, Wollersheim H. Planning and organizing the change process. In: Grol R, Wensing M, Eccles M, Davis D, editors. Improving patient care: the implementation of change in health care. 2nd ed. Chichester, UK: wiley-Blackwell; 2013. p. 64–74.

[17] Carron T, Rawlinson C, Arditi C, et al. An overview of reviews on interprofessional collaboration in primary care: effectiveness. Int J Integr Care. 2021;21(2):31. doi: 10.5334/ijic.5588. 34220395 PMC8231476

[18] European Forum for Primary Care. Position paper: organization of primary care. European Forum for Primary Care; 2022.

[19] Gabel S. Expanding the Scope of Leadership Training in Medicine. Acad Med. 2014;89(6):848–852. doi: 10.1097/ACM.0000000000000236. 24662199

[20] Mitterlechner M. Leadership in integrated care networks: a literature review and opportunities for future research. Int J Integr Care. 2020;20(3):6. doi: 10.5334/ijic.5420. PMC7427680 32863804

[21] Nieuwboer MS, Van Der Sande R, Van Der Marck MA, et al. Clinical leadership and integrated primary care: a systematic literature review. Eur J Gen Pract. 2019;25(1):7–18. doi: 10.1080/13814788.2018.1515907. 30474447 PMC6394325

[22] Braut H, Øygarden O, Storm M, et al. General practitioners’ perceptions of distributed leadership in providing integrated care for elderly chronic multi-morbid patients: a qualitative study. BMC Health Serv Res. 2022;22(1):1085. doi: 10.1186/s12913-022-08460-x. 36002824 PMC9404619

[23] Spehar I, Sjøvik H, Karevold KI, et al. General practitioners’ views on leadership roles and challenges in primary health care: a qualitative study. Scand J Prim Health Care. 2017;35(1):105–110. doi: 10.1080/02813432.2017.1288819. 28277051 PMC5361414

[24] Grol S, Molleman G, van Heumen N, et al. General practitioners’ views on the influence of long-term care reforms on integrated elderly care in the Netherlands: a qualitative interview study. Health Policy. 2021;125(7):930–940. doi: 10.1016/j.healthpol.2021.04.011. 33975761

[25] Merx LMH, Dijk-de Vries A, Veenstra MYM, et al. Towards an informal leadership framework for interprofessional primary care: an interview study among healthcare leadership experts. Research Square; 2025. Preprint.

[26] Braun V, Clarke V. Using thematic analysis in psychology. Qual Res Psychol. 2006;3(2):77–101. doi: 10.1191/1478088706qp063oa.

[27] Kippist L, Fitzgerald A. Organisational professional conflict and hybrid clinician managers: the effects of dual roles in Australian health care organizations. J Health Organ Manag. 2009;23(6):642–655. doi: 10.1108/14777260911001653. 20020597

[28] De Brún A, O’Donovan R, McAuliffe E. Interventions to develop collectivistic leadership in healthcare settings: a systematic review. BMC Health Serv Res. 2019;19(1):72. doi: 10.1186/s12913-019-3883-x. 30683089 PMC6347820

[29] Nieuwboer M, Van der Sande R, Olde Rikkert M, et al. Clinical leadership training in integrated primary care networks: a qualitative evaluation. Integr Healthc J. 2022;4(1):e000086. doi: 10.1136/ihj-2021-000086. 37440863 PMC10241027

[30] Oostra DL, Nieuwboer MS, Melis RJF, et al. DementiaNet facilitates a sustainable transition toward integrated primary dementia care: a long‐term evaluation. Alzheimers Dement. 2023;19(12):5498–5505. doi: 10.1002/alz.13154. 37218358

[31] Oostra DL, Harmsen A, Nieuwboer MS, et al. Care integration in primary dementia care networks: a longitudinal mixed-methods study. Int J Integr Care. 2021;21(4):29. doi: 10.5334/ijic.5675. PMC8663750 34963758

[32] El-Awaisi A, Yakti OH, Elboshra AM, et al. Facilitators and barriers to interprofessional collaboration among health professionals in primary healthcare centers in Qatar: a qualitative exploration using the “Gears” model. BMC Prim Care. 2024;25(1):316. doi: 10.1186/s12875-024-02537-8. 39192182 PMC11348528

[33] Green BN, Johnson CD. Interprofessional collaboration in research, education, and clinical practice: working together for a better future. J Chiropr Educ. 2015;29(1):1–10. doi: 10.7899/JCE-14-36. 25594446 PMC4360764

[34] Hennink M, Kaiser BN. Sample sizes for saturation in qualitative research: a systematic review of empirical tests. Soc Sci Med. 2022;292:114523. doi: 10.1016/j.socscimed.2021.114523. 34785096

