# Supplementary material for: Beyond clinical care: A qualitative study on the leadership role of general practitioners in dutch interprofessional care for older adults
Source: Eur J Gen Pract. 2026 Aug 26;32(1):2715197. doi: 10.1080/13814788.2026.2715197 (PMC13520846; doi:10.1080/13814788.2026.2715197)
Supplement: Supplementary file 2 [file IGEN_A_2715197_SM7156.docx]

**Appendix 1**

**Interview Guide – General Practitioners and Leadership in Interprofessional Care for Frail Older Adults**

**1. Background Information**

1. Could you describe your experience as a general practitioner?
   - How long have you been practicing?
   - Can you describe the type of practice you work in (solo, duo, or group practice)?
   - How would you characterise the location of your practice (urban, rural, other)?
2. How is care for frail older adults organised in your practice?
   - Could you describe the involvement of interprofessional teams? How formal or informal are these teams?
   - How is continuity of care maintained, and what is the typical composition of these teams?
   - How do your colleagues work together within these teams?

**2. Interprofessional Collaboration**

3. Thinking about recent interprofessional meetings you have attended, how would you describe your role in collaboration for older adults at the team level?
   - How do you perceive your role compared to that of your colleagues?
   - Can you describe situations in which someone else might take on leadership responsibilities?
   - How are roles and responsibilities discussed or clarified during team meetings?
   - How is accountability or ultimate responsibility expressed in discussions or in practice?
   - How are visions, goals, or team processes addressed and monitored?
4. In your experience, what personal qualities, behaviours, or skills support effective interprofessional collaboration?
   - Which of these are innate or personal, and which are learned?
   - How do you see leadership expressed in practice, for example through serving as a role model, discussing shared values or goals, or stimulating team members intellectually?

**3. Strengths and Challenges**

5. Based on your experience, what aspects of interprofessional collaboration currently work well in caring for frail older adults?
   - What factors contribute to these successes?
   - Who plays a key role in facilitating this collaboration?
   - What methods or strategies are used to achieve effective collaboration?
   - How is care managed when the GP is absent?
6. What challenges or difficulties do you observe in interprofessional collaboration?
   - Can you give examples of situations that did not work as expected?
   - What factors do you think contribute to these challenges?

**4. Impact on Patient Care**

7. How do you perceive the impact of team collaboration on patient care for frail older adults?
   - How do patients experience continuity of care?
   - How aware are patients of who is managing their care, and whom to contact for different issues?
   - Are there examples where work is duplicated or where tasks are not addressed?
8. Where do you see opportunities for improvement in interprofessional collaboration?
   - How might these improvements be implemented in practice?

**5. Closing**

9. Are there any additional themes, experiences, or insights you think are important to share regarding interprofessional collaboration or leadership in caring for frail older adults?
